# Elderly may benefit more from motor imagery training in gaining muscle strength than young adults: A systematic review and meta-analysis

**DOI:** 10.3389/fpsyg.2022.1052826

**Published:** 2023-01-04

**Authors:** Xiao J. Liu, Sha Ge, Alberto Cordova, Zayd Yaghi, Bo Y. Jiang, Guang H. Yue, Wan X. Yao

**Affiliations:** ^1^College of Art, Beijing Sport University, Beijing, China; ^2^College of Sports Science, Tianjin Normal University, Tianjin, China; ^3^Department of Kinesiology, College for Health, Community, and Policy, The University of Texas at San Antonio, San Antonio, TX, United States; ^4^School of Public Health, Jilin Medical University, Jilin, China; ^5^Center for Mobility and Rehabilitation Engineering Research, Kessler Foundation, West Orange, NJ, United States; ^6^Rutgers New Jersey Medical School, Rutgers, The State University of New Jersey, New Brunswick, NJ, United States

**Keywords:** motor imagery practice, mental practice, movement imagery, muscle strength, performance, strength enhancement, aging

## Abstract

**Objective:**

The current review was aimed to determine the effectiveness of mental imagery training (MIT) on the enhancement of maximum voluntary muscle contraction (MVC) force for healthy young and old adults.

**Data sources:**

Six electronic databases were searched from July 2021 to March 2022. Search terms included: “motor imagery training,” “motor imagery practice,” “mental practice,” “mental training,” “movement imagery,” “cognitive training,” “strength,” “force,” “muscle strength,” “performance,” “enhancement,” “improvement,” “development,” and “healthy adults.”

**Study selection and data extraction:**

Randomized controlled trials of MIT in enhancing muscle strength with healthy adults were selected. The decision on whether a study met the inclusion criteria of the review was made by two reviewers independently. Any disagreements between the two reviewers were first resolved by discussion between the two reviewers. If consensus could not be reached, then it would be arbitrated by a third reviewer.

**Data synthesis:**

Twenty-five studies including both internal MIT and external MIT were included in meta-analysis for determining the efficacy of MIT on enhancing muscle strength and 22 internal MIT were used for subgroup analysis for examining dose-response relationship of MIT on MVC.

**Results:**

MIT demonstrated significant benefit on enhancing muscle strength when compared with no exercise, Effect Size (ES), 1.10, 95% confidence interval (CI), 0.89–1.30, favoring MIT, but was inferior to physical training (PT), ES, 0.38, 95% CI, 0.15–0.62, favoring PT. Subgroup analysis demonstrated that MIT was more effective for older adults (ES, 2.17, 95% CI, 1.57–2.76) than young adults (ES, 0.95, 95% CI, 0.74–1.17), *p* = 0.0002, and for small finger muscles (ES, 1.64, 95% CI, 1.06–2.22) than large upper extremity muscles (ES, 0.86, 95% CI, 0.56–1.16), *p* = 0.02. No significant difference was found in the comparison of small finger muscles and large lower extremity muscles, *p* = 0.19 although the ES of the former (ES, 1.64, 95% CI, 1.06–2.22) was greater than that of the later (ES, 1.20, 95%, 0.88–1.52).

**Conclusion:**

This review demonstrates that MIT has better estimated effects on enhancing MVC force compared to no exercise, but is inferior to PT. The combination of MIT and PT is equivalent to PT alone in enhancing muscle strength. The subgroup group analysis further suggests that older adults and small finger muscles may benefit more from MIT than young adults and larger muscles.

## Introduction

Motor imagery (MI) is a phenomenon of internally perceiving or simulating an actual motor action without physically executing it by using motor-related parts of the brain as its substrate (Decety, 1996; Krüger et al., 2020). That is, MI is an active cognitive process during which the representation of a specific action is internally reproduced within working memory without any overt motor activities. Imaging studies show that MI increases activity in cortical areas that overlap with cortical areas activated during the actual performance of motor acts, providing evidence for the common coding between perception and action (Hinshaw, 1991; Decety, 1996; Decety and Grezes, 1999; Hétu et al., 2013). Repetitive application of MI in the acquisition of motor skills is MI training (MIT), which is also known as mental practice or action-imagery practice (Driskell et al., 1994; Simonsmeier et al., 2021; Dahm et al., 2022). MIT has shown to be effective in motor skill acquisition (Feltz and Landers, 1983; Driskell et al., 1994; Land et al., 2016; Lindsay et al., 2021). Additionally, MIT has also been reported to be beneficial to the elderly population, not only in motor performance but cognitive function as well (Altermann et al., 2014). Furthermore, studies (Shelton and Mahoney, 1978; Gould et al., 1980; Tod et al., 2015) also demonstrated that applying MI to or “Psyching-Up” during muscle contractions could improve muscle force outcome.

To examine the effect of MIT on muscle strength, Yue and Cole (1992) conducted one of the first investigations showing the efficacy of MIT enhancing maximal voluntary muscle force. The study by Yue and Cole (1992) included three groups (MIT, physical training, and control groups) and examined not only the MIT effect on improving muscle strength but also neuromuscular mechanisms underlying the MIT-induced strength increase. The young-healthy subjects trained their left hypothenar (little finger abductor) muscles for 4 weeks by either producing real maximal isometric contractions of the muscle (physical training group, PT), imagining producing these same isometric contractions (motor imaging training group, MIT), or having no training of the muscle at all (no-exercise control group, CTRL). After the 4-weeks training, the maximal little finger abduction force increased significantly for the MIT group and the PT group (22 and 30%, respectively), but not for the CTRL group (3.7%). Since MI is a process to internally simulate an actual motor action without physically executing it, it is not expected to have any changes in muscle fiber hypertrophy through repetitive rehearsal of MI of the maximum voluntary muscle contraction (MVC) of the muscle. Muscle fiber hypertrophy has been shown to be one of the potential factors accounting for the muscle strength increases after physical resistance training (Jones et al., 2008; Krzysztofik et al., 2019). Alternatively, the authors (Yue and Cole, 1992) attributed the increase of muscle strength after the MIT to the increase of brain-to-muscle signal, that might lead to the recruitment of large motor units and an increase in discharge rate of originally recruited motor units in an MVC. Muscle force is controlled by the recruitment and discharge rate of motor units. Moreover, the central nervous system can increase muscle force by increasing the number of active motor units and the firing rate of the originally recruited motor units (Duchateau and Enoka, 2011; Enoka and Pearson, 2014). It has been shown that large-sized motor units cannot be voluntarily activated by the untrained young (Yue et al., 2000) and old adults (Yue et al., 1999; Rozand et al., 2020). However, as Yue and Cole (1992) suggested, MIT could significantly enhance brain-to-muscle drive that in turn, might help recruit the larger motor units, which were otherwise inactive in an untrained state, and drive the originally activated motor units to higher discharge rate, therefore, leading to greater muscle force. This finding has significant application in rehabilitation medicine (Jackson et al., 2001) since weak patients or frail older adults, who find it difficult or unsafe to participate in conventional strength training programs, may be able to enhance their muscle strength *via* their mind.

Since the seminal work by Yue and Cole (1992), the efficacy of MIT on MVC muscle force has been tested with different muscles and types of muscle contractions (Ranganathan et al., 2004; Sidaway and Trzaska, 2005; Wright and Smith, 2009; de Ruiter et al., 2012), in exploring the functional role of internal and external MIT in muscle strength enhancement (Yao et al., 2013; Montuori et al., 2018), on determining its effect on muscle strength in aging population (Darvishi et al., 2013; Mamone, 2013; Jiang et al., 2016; Goudarzian et al., 2017) and on its role in maintaining or enhancing the MVC force for the individuals with temporary immobilization of the upper extremity or anterior cruciate ligament (Yue et al., 1996; Slimani et al., 2016). There are two common types of mental imagery—internal and external imagery. In internal imagery (IMI; also known as kinesthetic or first-person imagery), a person imagines or mentally creates the feeling of performing the exercise from within the body (i.e., from a first-person perspective). For example, mental strength training using internal imagery emphasizes that the subject generates a similar feeling as he/she felt during a physical MVC (Ranganathan et al., 2004; Sidaway and Trzaska, 2005; Yao et al., 2013). In external imagery (EMI; also known as third-person visual imagery), the person sees or visualizes performing the task from outside the body—similar to watching oneself in a mirror performing an exercise (Herbert et al., 1998). Performing IMI generates significantly more physiological responses (i.e., heart rate, blood pressure, and respiration rate) compared to doing EMI (Ranganathan et al., 2004) and is superior to EMI for elevating MVC-related cortical potentials on scalp locations over primary motor (M1) and supplementary motor cortices and for enhancing muscle strength (Yao et al., 2013).

Overall, accumulating evidence suggests that MIT is effective in enhancing muscle strength. While the majority of studies in the area presented the beneficial effect of MIT in enhancing muscle strength, contradictory findings were also demonstrated from a few studies, including a recent meta-analysis study (Manochio et al., 2015). In their study, Manochio et al. (2015) revealed an non-beneficial effect of MIT over no-exercise CTRL [ES (effect size) = −0.10, 95% CI (confidential interval) = −1.46 to 1.24]. However, as Paravlic et al. (2018) argued, the contradictory finding from study Manochio et al.’s (2015) could be due to its lack of statistical power since only four studies were included in the meta-analysis while many other relevant studies were available. It is possible that Manochio et al. (2015) only included studies with post-innervation values (PIV) and excluded studies with change-from-baseline values (CBV) due to the concerns about biased outcome (Cuijpers et al., 2017). However, it has been suggested that an analysis based on CBV could be more efficient and powerful than comparison of PIV, as it removes a component of between-person variability from the analysis (Durg et al., 2015; Deeks et al., 2022). It is further stated that it is statistically legitimate for a meta-analysis to include studies with both CBV and PIV (da Costa et al., 2013; Deeks et al., 2022; Higgins et al., 2022).

A more recent and outstanding systematic review and meta-analysis study by Paravlic et al. (2018), which included more relevant articles (13 articles published either in English or German by April 2017), demonstrated medium effect size (ES = 0.72, 95% CI = 0.45–0.97), based on the revised Cohen’s d effect size chart (Sawilowsky, 2009), in favor of the MIT groups over the no-exercise CTRL groups. The meta-analysis in study Paravlic et al.’s (2018) generated four forest plots, MIT vs. no-exercise CTRL, PT vs. no-exercise CTRL, MIT vs. PT, and MIT + PT vs. PT. In addition, the Meta-regression analysis showed that two out of six training volume variables, the number of repetitions per training session (*p* = 0.01) and per study (*p* = 0.05), predicted the effects of MIT on muscle strength enhancement and additional dose–response analysis further showed that the largest effects were found after the use of the greatest number of repetitions (Paravlic et al., 2018). Due to the limited number of available studies in old adults at the time, age was not included as a moderator variable in their meta-analysis (Paravlic et al., 2018). In an attempt to overcome this limitation, Meier (2021) conducted a systematic review and meta-analysis including both young and old adults. Consistent with the study of Paravlic et al. (2018), the meta-analysis by Meier (2021) also revealed a medium effect size (ES = 0.76, 95% CI = 0.53–0.99) for the young adults when the MIT groups were compared with the no-exercise CTRL groups. However, the MIT did not show clear beneficial effect for the old adults in study Meier’s (2021). A qualitative visual analysis of the studies’ results for the elderly group suggests a non-significant effect since the “gray rhombus,” reflecting the elderly group’s point estimate of the treatment effect, touched the line of no effect (ES = 0.53, 95% CI = −0.03–1.10) (Meier, 2021). The results of study Meier’s (2021) on older adults was surprising since previous studies (Sale, 1986, 1987; Semmler and Enoka, 2000; Simoneau et al., 2006; Mamone, 2013) suggest that elderly should be able to benefit from MIT as much as, if not more than, younger adults due to the greater neural deficit for the elderly that gives a larger potential for mental training-related strength improvements. The results of Meier’s study on older adults could be due to its use of PIV only and lack of the number of studies included. Indeed, all the three studies with older adults included in Meier’s study reported positive increase from the baseline in MVC force after the MIT, ranging from 8.7 to 18.6% (Table 2 in Meier’s study). Thus, the main objective of the current study was to undertake a systematic review and a meta-analysis of randomized controlled studies with either PIV or CBV available, published in peer-reviewed journals or unpublished dissertation or thesis, investigating the beneficial and adverse effects of motor imagery training (MIT) on enhancing muscle strength for both healthy young and older adults. It was hypothesized that both young and older adults should benefit from MIT to enhance their muscle strength. The hypothesis was developed based on the facts that imagery abilities are sustained with aging (Saimpont et al., 2013) and sarcopenia, the age-related decrease in lean muscle mass (Siparsky et al., 2014), makes it feasible to augment old adults’ muscle strength by increasing net excitation of the motoneurons, and increasing activation of prime movers and inhibition of antagonists (Sale, 1987) as the results of MIT.

## Research methods

The current review followed the checklists and guidance on systematic reviews described in PRISMA Statement 2020 (Page et al., 2021).

### Eligibility criteria

The PICOS’s (population, intervention, comparison, outcome, and study design) recommendation was adopted to determine the eligibility (Amir-Behghadami and Janati, 2020).

#### Inclusion criteria

(1) Population: Healthy male and female adults aged at or above 18 years; (2) Intervention: The MIT could be given as an independent intervention or combined with PP. The MIT had to be given at least three training sessions in three separate days and included at least one CTRL group (no-excise group) and/or one physical training (PT) group; (3) Comparison: maximal voluntary contraction (MVC) force was compared between (a) the intervention type (i.e., MIT vs. no-exercise CTRL, MIT vs. PT, and MI + PT vs. PT alone), (b) the age groups (young and old adults), and (c) the muscle groups trained (larger muscle groups vs. smaller muscle groups); (4) Outcome: The outcome measures could be either PIV (the post-innervation values) or CBV (the change-from-baseline values) of MVC force. If both PIV and CBV are available, CBV would be selected for further analysis [see Higgins et al. (2022) for the rationals behind the decision, Deeks et al. (2022)]; and (5) Study design: randomized controlled trials published in peer-reviewed journals or non-published dissertation/thesis before March 30, 2022 that tested efficacy of MIT on muscle strength improvement.

#### Exclusion criteria

(1) studies written in languages other than English; (2) non-randomized studies or studies with unhealthy populations; and (3) studies without enough information for obtaining means and standard deviations of the two comparison groups (e.g., MIT and CTRL) to calculate ESs and/or determine dose–response relationship.

### Search sources and screening strategy

Computer-aided search was performed by the two researchers (XL and WY) using PubMed/Medline, ERIC, Web of Science, Google Scholar, ScienceDirect, and ProQuest. Key words used were: “motor imagery training,” “motor imagery practice,” “mental practice,” “mental training,” “movement imagery,” “strength,” “force,” “muscle strength,” “performance,” “enhancement,” “improvement,” and “healthy adults.” To find additional articles, the authors hand-searched reference lists of obtained articles (reference and author tracking). The search was terminated on March 15, 2022.

To insure effective and accurate search, the titles and abstracts were first analyzed by the two reviewers based on the pre-determined inclusion and exclusion criteria. Then, the full texts of the remaining papers that met the inclusion criteria were retrieved and thoroughly reviewed by the two reviewers to determine which articles to include in the meta-analysis. Any studies for which suitability was unclear were then reviewed by the two reviewers (XL and WY) again and a decision was made through discussion between the two reviewers. If consensus could not be reached, then it would be arbitrated by a third reviewer (GY).

### Selection process

**FIGURE 1:**
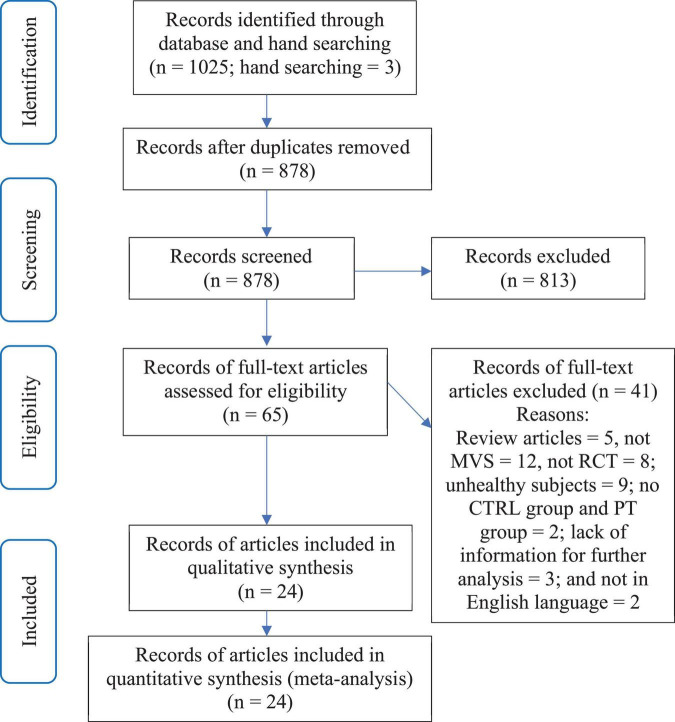
Study selection process.

As described above, the decision on whether a study met the inclusion criteria of the review was made by two reviewers independently (XL and WY). Any disagreements between the two reviewers were first resolved by discussion between the two reviewers. If consensus could not be reached, then it would be arbitrated by a third reviewer (GY). See Figure 1 for a more visible flow of selection process.

### Data extraction

Data were extracted by one reviewer (XL) into a data extraction spreadsheet that was developed based on the Template for Intervention Description and Replication checklist (Hoffmann et al., 2014). Data extraction was checked by a second reviewer (WY). The following data were extracted: study design; number of groups; sample size in each group; description of the intervention; randomization; frequency of MIT sessions; duration or number of trials of each MIT session; the length of the entire MIT intervention; and baseline and post-intervention values. When the manuscript did not present data needed for further analysis, the authors were contacted for this information. For the studies that data were shown in figures, the WebPlotDigitizer software (version 4.5, August, 2021; Ankit Rohatgi; Pacifica, CA, USA) was used to extract the necessary data.

### Risk of bias (methodological quality) assessment

Two reviewers (XL and WY) independently assessed the quality of all included studies using the PEDro criteria scores (de Morton, 2009). Three ratings were determined based on the scores: poor (< 2), moderate (3–5), or high (6–10) quality trials (de Morton, 2009). Any disagreements between the two reviewers were first resolved by discussion between the two reviewers. If consensus could not be reached, then it would be arbitrated by a third reviewer (GY).

### Reporting bias

Funnel plot was used to visualize evidence of publication bias and Egger’s regression test was performed to provide further statistical evidence of publication bias if any. The authors gave their views on the potential sources of the asymmetry and on the implications of the missing results (Page et al., 2022).

### Synthesis

Studies for the synthesis were selected based on the pre-determined inclusion and exclusion criteria and the outcomes of the selected studies were grouped based on their innervation types (i.e., MIT, PT, MIT + PT, and CTRL). The meta-analyses were performed using R-Studio (1.4.1717-3, “Juliet Rose” for macOS), a language and environment for statistical computing and graphics. Main packages used in the study were “meta,” “rmeta,” and “metafor.” Meta-analysis of the different continuous measures of MVC force, presenting results as point estimates and 95% confidence intervals (CI), was undertaken by one reviewer (WY). SMD (standardized mean differences) was used in the study since the outcomes in the study included both PIV (post-innervation values) and CBV (change-from-baseline values). Heterogeneity was assessed through visual inspection of forest plots and the calculation of the chi-square and *I*^2^ statistics. Subgroup analysis was conducted to examine the similarities and/or differences in MIT effect on MVC force between the two age groups (i.e., young vs. old adults) and three muscle groups (i.e., lower extremity, upper extremity, and finger muscles). In addition, the dose-response relationship of MIT effect on MVC force was also examined. Due to the concern of multi-collinearity among training variables such as training periods and total training trials and non-linear-relationship between estimated effects (dependent variable) and training variables (independent variables) that might pose a big threat to the validity of applying multiple meta-regression (Harrer et al., 2022), the dose-response relationship was examined by detecting the estimated effects of each training volume variable on the MVC force, instead of applying multiple meta-regression. The moderators in the dose-response analysis were the four training variables (training periods in weeks, training sessions per week, training trials per session, and total training trials per study). The *Q*-test based on the overall subgroup results was used to determine if the subgroup differed significantly (Harrer et al., 2022).

### Effect measures

Once again, meta-analysis of the different continuous measures of MVC force, presenting results as point estimates and 95% confidence intervals (CI), was undertaken by one reviewer (WY) and SMD (standardized mean differences) was used in the study since the outcomes in the study included both PIV (post-innervation values) and CBV (change-from-baseline values).

## Results

### Study selection

A total of 1,025 articles were identified by the online database literature search (Figure 1). Following the removal of duplicates and the elimination of articles based on title and abstract screening, 65 studies remained. An evaluation of the remaining 65 studies was conducted independently by the two reviewers (XL and WY). Following the final screening process, 24 articles involving 25 studies were included in the systematic review and meta-analysis.

### Risk of bias (methodological quality) assessment

PEDro scores are displayed in Table 1. Overall, all the included studies were rated with high quality (PEDro scores of 6.00). All the included studies received points for the following items: baseline indicators, obtaining measures of at least one key outcome from more than 85% of the subjects, allocation of all subjects in either a treatment or control condition, and reporting the results of statistical comparison between groups and measures of variability. On the other hand, all the included studies failed to satisfy the following items: concealed allocation, and blinded assessors/experimenters. All but one (Zijdewind et al., 2003) failed to report blinding of subjects.

**TABLE 1:** Characteristics and major outcomes of the included studies.

Articles	Pedro score	Age, mean ± SD	Sample size (N)	Characteristics of MIT	Weeks of MIT	Freq/ Weeks	T/S	TT of MIT	Key findings
Alenezi (2018)	6	Male and Female 24.43 ± 5.75	MIT = 16 MIT + VI = 16 CTRL = 15	Hip abduction; isometric	6	5	30	900	MIT 5.5% MIT+VI 6.7%*% CTRL NC
Bahari et al. (2011)	6	Male only 22.5 ± 1.36	MIT = 8 CTRL = 8	Elbow flexion; isometric	4	5	50	1000	MIT 30% * CRTL 5.5%
Bouguetoch et al. (2021)	6	Male and Female 24 ± 5.8	MIT = 10 CTRL = 7	Right plantar flexor muscles; Isometric	2	5	40	400	MIT 13.8%* CTRL NC
Cornwall et al. (1991)	6	Female only 21–25	MIT = 12 CTRL = 12	Knee extension; isokinetic	1	3	N/A	N/A	MIT 12.6%* CTRL 0.89%
Darvishi et al. (2013)	6	Male only 70.93	MIT = 10 PT = 10 CTRL = 10	Hand flexors; isometric	3	5	30	450	MIT 11.2%* PT 25%** CRTL 2.82%
de Ruiter et al. (2012)	6	Male and Female 18–24	MIT = 10 PT = 9 CTRL = 10	Leg extensors; isometric	4	3	10	120	MIT 9.3%* PT 6.6%* CTRL NC
Fontani et al. (2007)	6	Male only 35 ± 8.7	MIT = 10 PT = 10 CTRL = 10	Karate ridge hand strike dynamic	4	5	120	2400	MIT 9.2%** PT 8.4%** CRTL NC
Goudarzian et al. (2017)	6	Male and Female 68 ± 5.78	MIT = 12 PT = 11 CTRL = 9 MITPT = 10	Leg extension; isometri	8	5	30	1200	MIT 18.6%* PT 15.4%* CTRL 3% MITPT 23.8**
Grôspretre et al. (2017)	6	Male and Female 19–26	MIT = 9 CTRL = 9	Plantar flexion; isometric	1	7	100	700	MIT 9.64%* CTRL NC
Herbert et al. (1998)	6	Male and Female Young	EMIT = 18 PT = 18 CTRL = 18	Elbow flexion; isometric	8	3	6	144	MIT 6.5%* PT 17.8% ** CTRL 6.5%*
Jiang et al. (2016)	6	Male and Female 75 ± 7.9	MIT = 10 PT = 10 CTRL = 7	Elbow flexion; isometric	12	5	50	3000	MIT 26.1% PT 28.6% CTRL NC
Jiang et al. (2017)	6	Male and Female 18–35	HMIT = 6 LMIT = 6 CTRL = 6	Elbow flexion; isometric	6	5	30	900	HMIT 8.33%* LMIT 0.96% CG 2.61%
Lebon et al. (2010)	6	Male and Female 19.75 ± 1.72	MIT = 9 PT = 10	BP and LP dynamic	4	3	100	1200	MIT BP 9.37%** MIT LP 26.28%** PT BP 12.2%**
Leung et al. (2013)	6	Male and Female 18–35	MIT = 6 PT = 6 CTRL = 6	Elbow flexion; dynamic	3	3	32	288	MIT 16% PT 39% CTRL NC
Mamone (2013)	6	Male and Female 74.41 ± 7.09	MIT = 15 PT = 10 CTRL = 7	Elbow flexion; Isometric	8	5	30	1200	MIT 8.7% PT 8.4 CTRL-7%
Niazi et al. (2014)	6	Male only 22.4 ± 1.25	MIT = 16 PT = 16 CTRL = 15	Plantar flexors; isometric	4	5	50	1000	MIT 13.4%* CTRL 0.5%
Ranganathan et al. (2004)	6	Male and Female 24.43 ± 5.75	MITABD = 8 MITELB = 8 PT = 6 CTRL = 8	Finger abductor and elbow flexor; Isometric	12	5	50	3000	MITABD 35%** MITELB 13.5%** PTABD 53%** CTRLABD NC CTRLELB NC
Reiser et al. (2011)	6	Male and Female 22.7 ± 2.3	MIT25%PT = 12 MIT50%PT = 12 MIT75%PT = 12 PT = 12	BP and LP isometric	4	3	N/A	N/A	MIT25%PT 4.2% MIT50%PT 3.59% MIT75%PT 3.0% PT 5.1%
Shackel and Standing (2007)	6	Male only 18–24	MIT = 10 PT = 10 CTRL = 10	Hip flexors; hip flexor machine—dynamic movement	2	5	32	320	MIT 23.7%** PT 28.2%** CTRL 3.5%
Sidaway and Trzaska (2005)	6	Male and Female 19–26	MIT = 10 PT = 10 CTRL = 10	Ankle dorsiflexion; isokinetic	4	3	30	360	MIT 17.13%* PT 23.28%* CTRL 3.5% 1.77%
Smith et al. (2003)	6	Male and Female 24.43 ± 5.75	MIT = 16 PT = 16 CTRL = 15	Finger abduction; isometric	4	2	20	160	MIT 23.27%* PT 53.36%** CTRL 5.36%
Wright and Smith (2009)	6	Male and Female 20.74 ± 3.71	MIT = 10 PT = 10 CTRL = 10 EMIT = 10 MITPT = 10	bicep curl machine; dynamic	6	2	20	240	MIT 23.2%* PT 26.5%* CTRL 5.1% EMIT 13.7% MITPT 28%*
Yao et al. (2013)	6	Male and Female 18–35	MIT = 6 EMIT = 6 CTRL = 6	Elbow flexion; Isometric	6	5	30	900	MIT 10.5%* EMIT 4.8% CTRL NC
Yue and Cole (1992)	6	Male and Female 21–29	MIT = 10 PT = 8 CTRL = 9	Abduction of little finger of the hand	4	5	15	300	MIT 22.03%** PT 29.75% ** CRTL 3.7%

BP, bench press; CTRL, control group; Freq/WK, number of MIT sessions per week; LP, leg press exercise; MIT, motor imagery training; MITABD, MIT with little finger abductor muscle; MITELB, MIT with elbow flexor muscles; N/A, not available; NC, no change; PT, physical training; SD, standard deviation; T/S, number of trials per training session; TT/MIT, total trials in whole study period. *Indicates significant increase (*p* ≤ 0.05), **Indicates significant increase (*p* ≤ 0.01).

**TABLE 2:** Training volume variables with the effect size (ES): Motor imagery training* vs. no-exercise control.

Volume variables	Number of studies	Effect size (CI)	Heterogeneity			
			Q	df	*p*	I^2^
Training period (weeks)	22		58.86	24	<0.001	59.2%
3	6	1.52 (1.08–1.96)	2.52	5	=0.77	0.0%
4	7	1.16 (0.78–1.55)	6.00	6	=0.42	0.0%
8	6	1.07 (0.65–1.49)	23.19	5	<0.01	78.4%
12	3	1.95 (1.23–2.68)	1.44	2	=0.49	0.0%
Training frequency (per week)	22		58.86	24	<0.001	59.2%
3	6	1.06 (0.62–1.49)	11.40	5	<0.05	41.7%
5	16	1.39 (1.13–1.66)	25.73	15	<0.05	56.1%
Number of trials (per session)	22		58.86	24	<0.001	59.2%
20	3	1.18 (0.55–1.80)	9.80 2	2	=0.17	43.7%
30–40	11	1.48 (1.15–1.81)	19.82	10	<0.05	49.5%
50	6	1.05 (0.64–1.46)	17.41	5	<0.05	65.5%
100–120	2	1.41 (0.68–2.14)	0.02	1	=0.90	0.0%
Total number of trials (per study)	22		58.86	24	<0.001	59.2%
144–300	5	1.26 (0.77–1.75)	4.06	4	=0.40	1.4%
600	6	1.25 (0.83–1.68)	11.48	5	<0.05	56.4%
1,200	7	1.19 (0.80–1.57)	18.36	6	<0.05	67.2%
2,400–3,000	4	1.75 (1.16–2.33)	2.30	3	=0.51	0.0%

*Including only the 22 internal MIT studies in the analysis.

### Reporting bias

**FIGURE 2:**
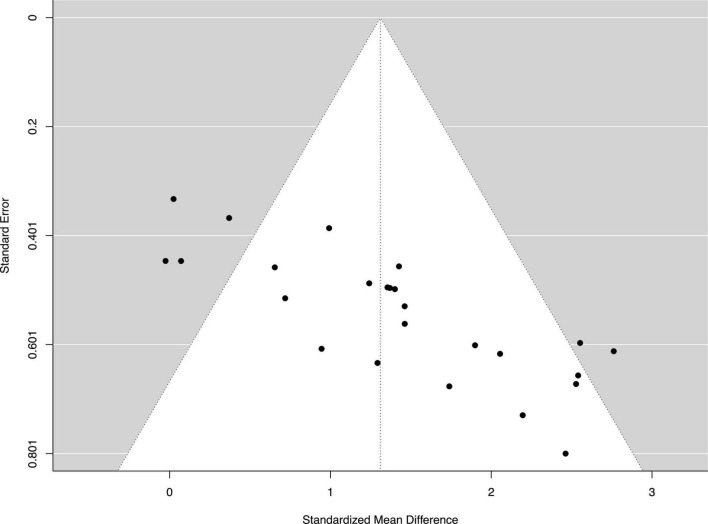
Funnel plot of the standard differences in means vs. standard errors. The aggregated standard difference in means is the random effects mean effect size weighted by the degrees of freedom study characteristics.

The funnel plot (Figure 2) suggests asymmetry for the publications. The Egger’s regression test provided further statistical evidence to show bias for the publications, *p* < 0.001.

After the online database literature search, 23 eligible articles with 25 MIT groups were selected (Table 1). Table 1 presents details of each included article regarding sample, measures, and key results. The sample size of the MIT groups in the included studies ranged from 6 to 18, three studies with a sample size of 6 (Leung et al., 2013; Yao et al., 2013; Jiang et al., 2017), two studies with a sample size of 8 (Ranganathan et al., 2004; Bahari et al., 2011), two studies with a sample size of 9 (Lebon et al., 2010; Grôspretre et al., 2017), nine studies with a sample size of 10 (Yue and Cole, 1992; Sidaway and Trzaska, 2005; Fontani et al., 2007; Shackel and Standing, 2007; Wright and Smith, 2009; de Ruiter et al., 2012; Darvishi et al., 2013; Jiang et al., 2016; Bouguetoch et al., 2021), three studies with a sample size of 12 (Cornwall et al., 1991; Reiser and Munzert, 2011; Goudarzian et al., 2017), one study with a sample size of 15 (Mamone, 2013), three studies with a sample size of 16 (Smith et al., 2003; Niazi et al., 2014; Alenezi, 2018), and one study with a sample size of 18 (Herbert et al., 1998). All the selected studies included a non-exercise and/or a non-imagery control group.

Fourteen selected studies included an additional PT group (Yue and Cole, 1992; Herbert et al., 1998; Smith et al., 2003; Ranganathan et al., 2004; Sidaway and Trzaska, 2005; Fontani et al., 2007; Shackel and Standing, 2007; Wright and Smith, 2009; de Ruiter et al., 2012; Darvishi et al., 2013; Leung et al., 2013; Mamone, 2013; Niazi et al., 2014; Jiang et al., 2016; Goudarzian et al., 2017). Four studies examined the effect of MIT on older subjects (Darvishi et al., 2013; Mamone, 2013; Jiang et al., 2016; Goudarzian et al., 2017). Most of the included studies investigated the effects of internal MIT, but three of the studies either examined both internal MIT and external MIT (EMIT) (Wright and Smith, 2009; Yao et al., 2013) or examined the effect of EMIT only (Herbert et al., 1998). The selected studies varied in training volume, ranging from 1 to 12 weeks, 3 to 7 sessions per week, and 6 to 120 trials per session (Table 1). In addition, the selected studies varied also in muscles examined, nine studies trained lower extremity (LE) muscles (Cornwall et al., 1991; Sidaway and Trzaska, 2005; Shackel and Standing, 2007; de Ruiter et al., 2012; Niazi et al., 2014; Goudarzian et al., 2017; Grôspretre et al., 2017; Alenezi, 2018; Bouguetoch et al., 2021), ten studies trained upper extremity (UE) muscles (Herbert et al., 1998; Ranganathan et al., 2004; Fontani et al., 2007; Wright and Smith, 2009; Bahari et al., 2011; Leung et al., 2013; Mamone, 2013; Yao et al., 2013; Jiang et al., 2016, 2017), and four studies trained finger muscles (Yue and Cole, 1992; Smith et al., 2003; Ranganathan et al., 2004; Darvishi et al., 2013).

### Overall findings of effects of motor imagery training on maximal voluntary contraction

#### MIT effects compared with no training (CTRL)

##### Overall estimated effects

**FIGURE 3:**
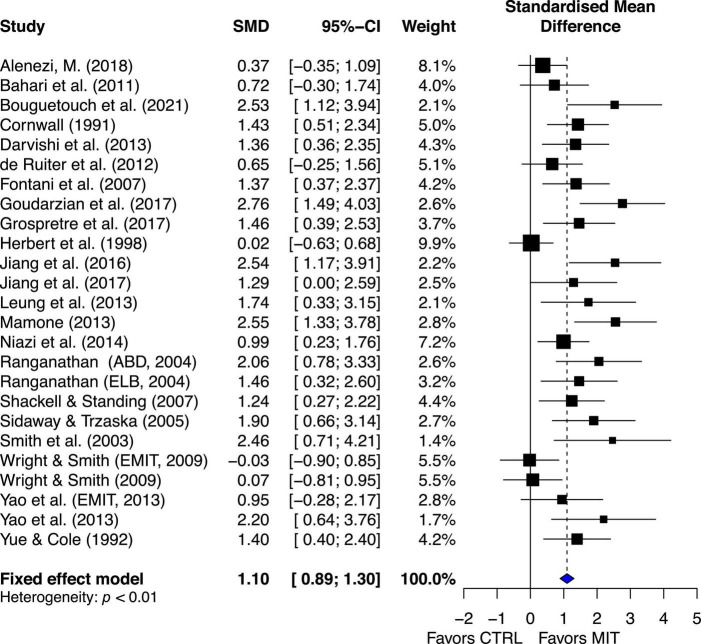
Effects on maximal muscle strength: Mental imagery training (MIT) vs. control group (CTRL).

Compared to the CTRL, the estimated effect of MIT was moderately beneficial for enhancing MVC force (ES = 1.10, 95% CI 0.89–1.30) (Figure 3).

The chi-square test for heterogeneity was significant (Q_24_ = 58.86, *p* < 0.01). The *I*^2^ value was 59%, indicating medium heterogeneity (Higgins et al., 2003). The results suggest there was study variability/heterogeneity among all the included studies.

##### Aging subgroup estimated effects

**FIGURE 4:**
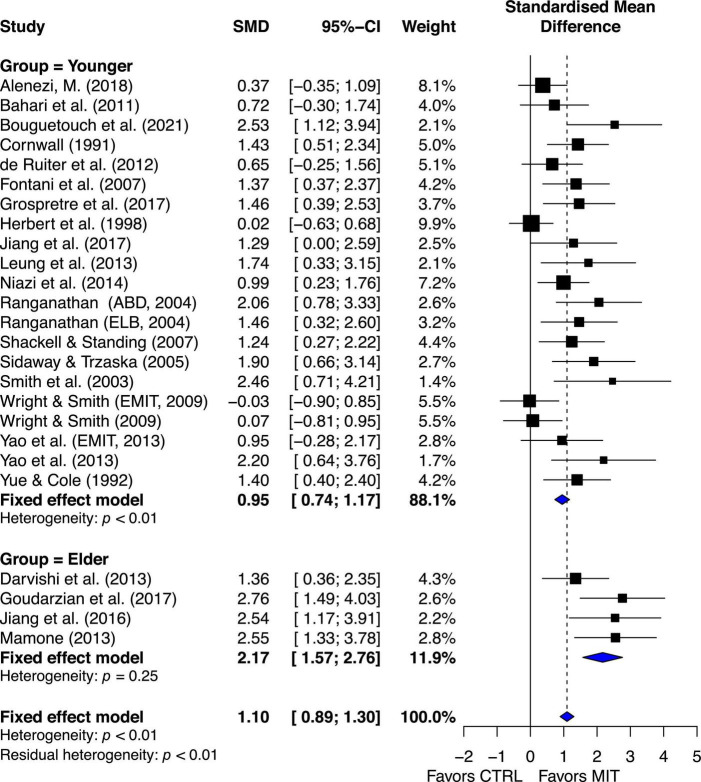
Aging subgroup’s effects on maximal muscle strength: Mental imagery training (MIT) vs. control group.

The estimated effect of MIT was more beneficial for the elder group than for the younger group in enhancing muscle strength, ES = 2.17, 95% CI = 1.57–2.76, and ES = 0.95, 95% CI = 0.74–1.17, respectively. Quantitative analysis provided further evidence to show significant statistical difference between the two groups, Q_1, 23_ = 14.15, *p* = 0.0002. A qualitative analysis of the younger-group studies’ results suggests between-study variability (Figure 4).

The chi-square test for heterogeneity was significant at a level of < 0.01. The *I*^2^ value was 50.8%, indicating medium heterogeneity (Higgins et al., 2003). These quantitative results suggest there was study variability/heterogeneity among the younger-group studies. In contrast, the chi-square test for heterogeneity in the elder-group studies was non-significant, *p* = 0.25. The *I*^2^ value was 26%, indicating small heterogeneity (Higgins et al., 2003). The results suggest that there was little between-study variability or heterogeneity in the aging studies.

##### Muscle subgroup estimated effects

**FIGURE 5:**
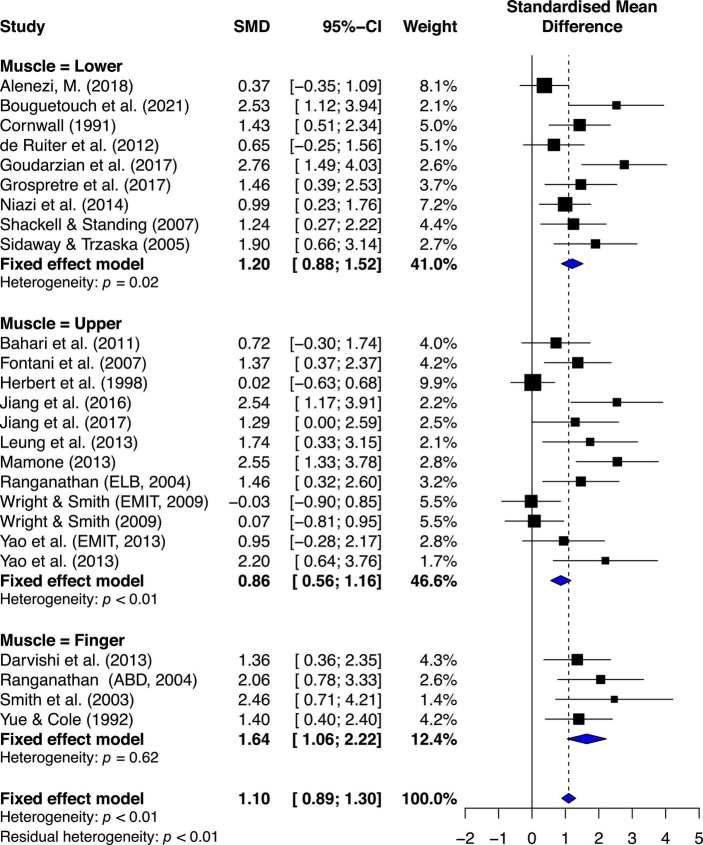
Muscle subgroup’s effects on maximal muscle strength: Mental imagery training (MIT) vs. control group.

The estimated effects of MIT for lower extremity (LE), upper extremity (UE), and finger muscles are ES = 1.20, 95% CI = 0.88–1.52; ES = 0.86, 95% CI = 0.56–1.16; and ES = 1.64, 95% CI = 1.06–2.22, respectively (Figure 5).

Quantitative analysis indicated significant statistical difference between the groups, Q_2, 22_ = 6.07, *p* = 0.0481. Further quantitative analyses of two-group comparisons on the effects of MIT indicated the only significant comparison between finger muscle and UE muscle, Q_1,14_ = 5.38, *p* = 0.02, and no other significant differences between finger muscle and LE muscle (Q_1,11_ = 1.65, *p* = 0.19), and between LE muscle and UE muscle (Q_1,19_ = 2.30, *p* = 0.12). A qualitative analysis of the younger-group studies’ results suggests between-study variability (see Figure 3). The chi-square test for heterogeneity was significant at a level of < 0.01. The *I*^2^ value was 50.8%, indicating medium heterogeneity (Higgins et al., 2003).

#### Motor imagery training effects compared with physical practice

**FIGURE 6:**
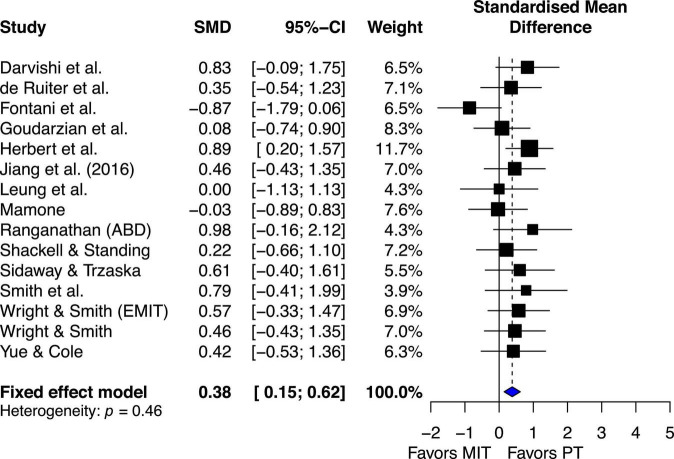
Effects on maximal muscle strength: Mental imagery training (MIT) vs. physical training (PT).

The estimated effect for the 15 studies demonstrated small beneficial effect (ES = 0.38, 95% CI 0.15–0.62), favoring PT. Although the qualitative visual analysis of the overall estimated effect for the 15 studies suggests possible heterogeneity (Figure 6), the chi-square test for heterogeneity was not significant, Q_14_ = 13.85, *P* = 0.46. The *I*^2^ value was 0%, indicating little heterogeneity (Higgins et al., 2003). These quantitative results suggest there was no significant study variability/heterogeneity among the studies that included both PT and MIT groups.

#### MIT combined with PT (MITPT) vs. PT only

**FIGURE 7:**
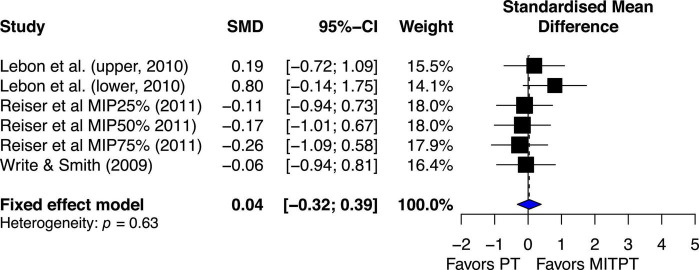
Effects on maximal muscle strength: Mental imagery training combined with physical training (MITPT) vs. physical training (PT).

Overall, the estimated effect of MITPT in enhancing MVC force was almost same as PT’s estimated effect (ES = 0.04, 95% CI −0.32–0.39) (Figure 7).

The chi-square test for heterogeneity was significant (Q_5_ = 3.48, *p* = 0.62). The *I*^2^ value was 0%, indicating no heterogeneity (Higgins et al., 2003).

#### Dose-response relationship of MIT effects on MVC force

The dose-response relationship was examined by detecting the estimated effects of each training volume variables on the MVC force (Table 2). The training period for the included studies ranged from 1 to 12 weeks, median = 4. The largest mean effect (ES = 1.95, 95% CI 1.23–2.68) was associated with a period of 12-weeks training (three studies only). The training sessions per week ranged from 2 to 7 (only one study with seven sessions per week), median = 5. The largest effect (ES = 1.39, 95% CI 1.13–1.66) was associated with the five sessions per week training frequency (16 studies). The number of trials per session ranged from 6 to 120, median = 32. The largest mean effect (ES = 1.48, 95% 1.15–1.81) was observed at the 30–40 trials per session (11 studies). The total trials per study ranged from 144 to 3,000, median = 700. The largest mean effect (ES = 1.75, 95% 1.16–2.33) was associated with the 3,000-trials per study (four studies). Although the differences in the values among the groups in each training variable were noticeable, they were not statistically significant, Q(3) = 5.66, *p* = 0.13; Q(1) = 1.68, *p* = 0.19; Q(3) = 2.80, *p* = 0.42; and Q(3) = 2.65, *p* = 45, for Training period, Training sessions per week, number of trials per session, and total number of trials per study, respectively.

## Discussion

The main purpose of this study was to conduct a systematic review and a meta-analysis of randomized controlled studies to examine the beneficial and adverse effects of motor imagery training (MIT) on enhancing muscle strength for both healthy young and older adults. The results of current study showed that MIT generates moderate improvements in muscle strength for both young and older adults (Figure 4). While the current study clearly presents beneficial effect of MIT, PT overperformed MIT in enhancing muscle strength (Figure 6) which is consistent with the findings of the previous studies (Paravlic et al., 2018; Meier, 2021). When the estimated effect of MIT was analyzed in subgroups based on the trained muscle, the results (Figure 5) indicated that finger muscles (ES = 1.64) benefited significantly more from MIT than the upper extremity muscles (ES = 0.86) but had no significant difference compared to the lower extremity muscles (ES = 1.20). In addition, a meta-regression analysis with the training volumes showed that only the number of sessions per week significantly predicted the effects of MIT on strength gain. However, it should be noted that there was moderate heterogeneity of the effects within each meta-analysis with comparisons of MIT and no exercise, suggesting that possible varied population effects were examined (Fletcher, 2007; Borenstein et al., 2009). However, it should be noted that the interpretation of the results of this study might be overestimated due to the publication bias (Figure 2).

### Overall effects of MIT on maximal voluntary contraction (MVC) force or muscle strength

Consistent with the previous reviews (Paravlic et al., 2018; Meier, 2021), the current review showed moderate overall estimated effect of MIT (ES = 1.10, 95% CI = 0.89–1.3) on enhancing muscle strength compared to no exercise groups (Figure 3). However, the results of both qualitative (Figure 3) and quantitative (Q_24_ = 58.86, *p* < 0.001, *I*^2^ = 59%) analysis of heterogeneity suggested the existence of study variability/heterogeneity among all the included studies. The estimated effect of variability among all the included studies could be caused by quality of MIT performed by subjects of different studies and/or different forms of MIT (i.e., internal MIT and external MIT) adopted by the analyzed studies. The Figure 3 shows that the estimated effects of two of the three studies (Herbert et al., 1998; Wright and Smith, 2009; Yao et al., 2013) falling on the no-effect line used external MIT (EMIT) while those of all other twenty-two studies falling on the right side of the no-effect line favoring MIT adopted internal MIT. This result was not surprising since studies (Ranganathan et al., 2004; Yao et al., 2013) have demonstrated that the effects of internal MIT were more effective than those of EMIT on improving the power of central drive [defined by motor-related cortical potential (MRCP) derived from scalp EEG data] that led to the enhancement of MVC force. Literature suggests that early strength gains are mainly due to neural adaptations including increases in the neural (descending) drive that leads to motor-unit recruitment and/or increases in firing rate of the activated motor unites (Semmler and Enoka, 2000), and/or changed input signals to the motoneuron pools that improve muscle coordination (e.g., reduction of antagonist muscle activation level during MVC, Mamone, 2013). Additional support for the effect of neural adaptations on muscle force is from the observation of the influence of motor-unit synchronization on muscle force. Previous studies (Milner-Brown et al., 1975; Semmler and Nordstrom, 1998) found that the level of motor-unit synchronization appears to be greater with hand muscles of individuals who consistently engage themselves in hand-muscle strength training, and one explanation of this finding is that an enhancement of motor-unit synchronization contributes to training-induced increases in muscle strength see Yao et al. (2000); Yao (2004), for different view on the effect of motor unit synchronization on muscle force.

While the current review demonstrated strong evidence for MIT effects on the enhancement of muscle strength when compared to no exercise, physical training still resulted in better mean effect (ES = 0.38, 95% CI 0.15–0.62) than MIT (Figure 6), that are consistent with the comparisons of MIT and PT in motor skill acquisition (Feltz and Landers, 1983). Favorable PT effect over MIT is not surprising since PT works on both muscle and central nervous system (CNS) (Sale, 1986; Enoka, 1997; Gandevia, 1999) but MIT works on CNS only (Ranganathan et al., 2004; Grôspretre et al., 2017). In general, the current review shows that PT is better than MIT. However, MIT is better than no exercise. This finding alone is important because it shows the efficacy of MIT in enhancing muscle strength which has great application in rehabilitation settings. An even more impressive finding from the current review is the effect of using a combination of PT and MIT. The combination of PT and MIT shows no noticeable difference in mean effect on enhancing MVC compared PT (Figure 7), ES = 0.04 favoring PT. One of the more extensive comparisons of combinations of MIT and PT was made in a study by Reiser and Munzert (2011). They compared five different PT and MIT conditions. One was 100% PT, and the three combinations were training routines requiring 75% PT and 25% MIT, 50% PT and 50% MIT, and 25% PT and 75% MIT. The control group required neither PT nor MIT. The results showed that the three combination groups demonstrated the increase of MVC force (3.0 to 4.2%) similar to the PT group (5.1%) but significantly greater than the control group (−0.2%). A noteworthy and interesting finding is that all three rates of MIT (25, 50, and 75%) yield nearly the same improvement of muscle strength as PT alone. This is inconsistent with a similar study by Hird et al. (1991), in which they compared the effect of PT alone with the effects of different levels of combination of PT and MIT on learning two motor skills. One was to learn a pegboard task and the other was to learn a rotary pursuit task. Regarding the effect of rates of MIT on learning the two motor skills, they (Hird et al., 1991) found that as the proportion of PT increased, the better learning would be. The discrepancy between the two studies could be explained by the difference in the tasks used in the two studies. The tasks examined in the current review were simple performances of muscle contractions but the tasks in the study by Yue and Cole (1992) were more complicated performances of motor skills. The development of performance of motor skills needs not only understanding of the movement but also visual, tactile, and proprioceptive feedback. It has been well-documented that MIT has an advantage in improving cognitive understanding of movement, and thus could facilitate the formation of the fundamental movement patterns, which is important during the early stages of learning (Driskell et al., 1994). However, imagery is not able to provide direct knowledge of results or visual and tactile feedback, which is critical for the improvement of performance during the later stages of motor skill learning. Thus, MIT might be as effective as PT during the early stage of learning when the understanding of movement sequences is a major concern but overall, PT might be superior to MIT due to its role in both obtaining knowledge of fundamental movement patterns and using visual, tactile, and proprioceptive feedback for refining motor skills (Driskell et al., 1994; Land et al., 2016).

### Aging subgroup effects of MIT based on MVC force

One of the objectives of the study was to compare the effect MIT on enhancing muscle strength for young adults and older adults. Previous review by Meier (2021) showed moderate estimated effects of MIT on strength for both older (ES = 0.53, 95% CI = −0.03–1.10) and younger (ES = 0.76, 95% CI = 0.53–0.99) adult. Since the “gray rhombus” reflecting the elderly group’s point estimate of the treatment effect touched the line of no effect, it was suggested that little or unclear beneficial effect of MIT on muscle strength for the older adults. However, as argued earlier, the results of Meier’s study on older adults could be due to its use of PIV only and a small number of studies included. When CBV was also used and one more study added, the current review demonstrated clear beneficial effect of MIT on strength for older adults (Figure 4). Indeed, the estimated effect of MIT was more beneficial for the elder group than for the younger group in improving muscle strength, ES = 2.17, 95% CI = 1.57–2.76, and ES = 0.95, 95% CI = 0.74–1.17, respectively, and the difference between the two groups was statistically significant, Q_1, 23_ = 14.15, *p* = 0.0002. In addition, both qualitative (Figure 4) and quantitative (Q_21_ = 40.64, *p* < 0.01, *I*^2^ = 50.8%) results suggest heterogeneity for the young adults. In contrast, such heterogeneity did not occur for the older adults (Q_4_ = 2.17, *p* = 0.25, *I*^2^ = 26.4%). The little heterogeneity for the older adults suggests that all the estimated points of treatment for the elderly individuals might come from the same population (Higgins et al., 2003).

Neural deficit, such as the number of motor units in the muscle that cannot be recruited by maximal voluntary effort, has been reported to be a major factor causing strength loss. For example, a study by Clark et al. (2006) investigated adaptations in neural control of the plantar flexor muscles after 4 weeks of unilateral lower limb suspension. Clark et al. (2006) found that, with regards to relative contribution of neural and muscular factors to strength loss, neural deficits in central activation explained 48% of the variability in strength loss, whereas muscular factors explained 39% of the variability. Central neural degeneration or neural deficit is a significant underlying mechanism of aging-related muscle weakness. It has been shown that neural deficit, for the elderly is greater than young adults (Campbell et al., 1973; Mamone, 2013). MIT, which increases central neural drive, may be a way to reverse the degeneration to improve strength. Therefore, it was suggested that MIT in elderly could reduce neural deficit by improving the cortical-to-muscle drive, therefore improving motor unit recruitment/activation and strength. The brain signal data of the study by Mamone (2013) showed that EEG frequency power associated with MVC of the elderly was significantly greater than young adults following an 8-week MIT. The EEG power has consistently shown a positive relationship with contractile force of a muscle group (Ranganathan et al., 2004; Mamone, 2013; Yao et al., 2013). Additionally, it has been shown that the level of functional connectivity between the brain (EEG) and muscle (EMG) signals increases with muscle force in old and young (Bayram et al., 2015), suggesting that the strength of the connectivity or coupling is important for muscle output. As it has been well-documented that the contractile force of a muscle is determined by the number of recruited motor units and the firing rate of the recruited motor units and both the recruitment and firing rate are modulated by commands from the central nervous system; the greater beneficial effect of MIT on muscle strength for elderly than young adults demonstrated in the current review might be explained at least partially by the greater EEG power for the elderly followed MIT (Mamone, 2013), and perhaps improved brain-to-muscle signal connectivity (Bayram et al., 2015) following the MIT.

### Muscle subgroup effects of MIT based on MVC force

Another interesting finding from the current review is that the estimated effect of MIT for finger muscles (ES = 1.64, 95% CI = 1.06–2.22) was significantly greater than the large UE muscles (ES = 0.86, 95% CI = 0.56–1.16), Q_1,14_ = 5.38, *p* = 0.02. This finding is consistent with the one from Ranganathan et al.’s (2004) study in which they directly compared MIT effects on the improvement of muscle strength of little finger abductor muscle (ABD group) and elbow flexor muscles (ELB group). After 12-weeks training, they (Ranganathan et al., 2004) found that the ABD group had increased their finger abduction strength by 35% (*P* < 0.005) and the ELB group augmented their elbow flexion strength by 13.5% (*P* < 0.001).

A potential explanation for the difference in ES of MIT between finger muscles and large muscles of the upper extremities may be due to the difference in the characteristics of motor unit behaviors between the two types of muscles. Literature indicates that muscle force is generated by increasing motor-unit recruitment and discharge rate of the recruited motor units. Studies (Milner-Brown et al., 1973; De Luca et al., 1982) have demonstrated that motor-unit recruitment is completed at about 40% of the MVC for small muscle groups such as finger muscles and at about 80% of the MVC for large muscle groups such as elbow flexor and knee extensor muscles. The force beyond these levels (∼40% of MVC for small muscles and ∼80% for large muscles) is produced purely by increasing discharge rate, which indicates that the central drive may have greater impact on firing rate of finger than larger limb muscles. In addition, outperformance of MIT of finger muscles over large upper extremity muscles may also be due to the relatively large cortical representation of the finger muscles, thus having greater potential to be influenced by MIT, over the large upper extremities. Furthermore, the large upper extremity muscles such as elbow flexor muscles are frequently used for daily activities and may be considered as “highly trained” with little room for neural adaptation-induced strength improvements. It should be noted that the magnitude of force production is directly proportional to the amplitude of the respective brain signal (Dai et al., 2001) and greater power of central motor control network has been observed following MIT (Ranganathan et al., 2004; Mamone, 2013; Yao et al., 2013). Thus, the greater effect of MIT on MVC force for the finger muscles might be explained by their greater representation in the brain (motor cortex). However, since there was no significant difference in ES of MIT between finger muscles (ES = 1.64) and large lower extremity muscles (ES = 1.20), additional factors must exist to account for the difference in the effect of MIT on these different muscle groups. Due to the lack of the literature, these factors cannot be identified and should be addressed in the future research.

### Dose-response relationship of MIT to MVC force

The dose-response relationship of MIT to MVC force was examined by detecting mean effects associated with training volume variables (see Table 2). For the training period, no significant difference between groups was found, Q(3) = 2.65, *p* = 0.45. It (Table 2) shows that 3-weeks or less training period yields mean MIT effect equivalent to those with longer training periods. It is not surprising for the 3-weeks or less training period of MIT yields a substantial improvement of MVC force since studies (Sale, 1986; Enoka, 1997; Chilibeck et al., 1998; Akima et al., 1999) have clearly shown that early strength gains within the first 3 to 4 weeks of a conventional strength training program are mainly attributed to the neural adaptations. Two studies with only one week’s MIT training (Cornwall et al., 1991; Grôspretre et al., 2017) also showed a significant increase in muscle strength compared to the baseline (see Table 1). In the 1-week training by Grôspretre et al. (2017), the MIT group increased both plantar flexion MVC torque and the rate of torque development (RTD) along with the significant increase of EMG activity and V-wave during MVC and of H-reflex at rest. Thus, Grôspretre et al. (2017) attributed the significant enhancement of MVC torque and RTD after 1-week MIT training to the increased cortical descending neural drive and the excitability of spinal networks at rest. In a longitudinal study, Ranganathan et al. (2004) inspected MVC force changes of both little finger abductor (ABD) and elbow flexor (ELB) muscles under MIT intervention over 12 weeks. The maximal gain of MVC was obtained after 4-week training for ABD and 2-week training for ELB. Furthermore, the enhancements of MVC force of both ABD and ELB groups were accompanied with the significant increase of MVC-related cortical potential. Putting together, there is no research evidence to suggest an optimal training period for MIT. It seems that the optimal training is muscle specific. The limited research evidence tentatively suggests that larger muscle groups need one to 2 weeks of MIT training to achieve significant improvement (Ranganathan et al., 2004; Grôspretre et al., 2017) and small finger muscles may need a longer training period, three to 4 weeks, to obtain maximal MIT effect (Ranganathan et al., 2004).

For the training sessions per week, 15 out of 22 studies used five sessions per week. Compared to the less frequent training (3 or 2 sessions per week), the more frequent training (5 or 7 sessions per week) shows a noticeable advantage but the difference between the two was not significant, Q(1, 20) = 1.68, *p* = 0.20. The estimated MIT effect for 5 or more sessions is ES = 1.39, 95% CI 1.13–1.66 and for 3 or 2 sessions is ES = 1.06, 95% CI 0.62–1.49. Due to the lack of the significant difference between the two groups, thus no recommendation could be made for the optimal training frequency in the current review and further research is needed to address the issue.

Regarding the number of trials per session, most of studies (11) had 30 to 40 trials in a session that also resulted in largest mean MIT effect (ES = 1.48, 95% CI 1.15–1.81). It should be noted that the difference in mean MIT effects among the number of trials per session groups is not significant, Q(3) = 2.80, *p* = 0.42. Thus, the decision of the number of trials per session should be made based on training period, number of sessions per week, and even individual’s imagery ability as suggested by Paravlic et al. (2018).

For the total number of trials per study, similar to the number of trials per session, the difference in mean MIT effects among the groups is not significant, Q(3) = 2.65, *p* = 45 and ES for the four groups are remarkably close, ranging from 1.19 to 1.75. Although no optimal total trails in a study could be identified, it should be noted that majority of the included studies had 600 or more total training trials. Thus, it is suggested that a minimum of number of 600 trials might be needed.

### Limitations of the current review

Like many other studies with systematic reviews and meta-analyses, the current review has some limitations. The funnel plot (Figure 2) is skewed asymmetrically indicating publication bias. The Egger’s regression test provided further statistical evidence to show bias for the publications, *p* < 0.001. The publication bias may lead to overestimation of the effectiveness of MIT. The cause of the publication bias may come from the fact that studies reporting statistically significant effect sizes are more likely to be published than studies reporting negative effect size (Samawi, 2021). In addition, inclusion of the articles published in English only may also be a factor to cause the publication bias. Additional limitation is from the lack of number of studies to achieve statistical power. This is true in our subgroup analysis to detect a dose-response relationship of MIT on muscle strength. Thus, it is difficult to identify optimal volumes in the four training variables (Table) examined in the current review because of the absence of statistical evidence (i.e., statistically not significant between subgroups). It should be noted that absence of evidence is not evidence of absence. In other words, no significant difference in effect sizes between subgroups does not automatically mean that the subgroups produce equivalent outcomes. A further limitation to the findings of this review is that no studies comprehensively examined the efficacy of MIT on enhancing muscle strength. In other words, the focus and design of MIT intervention is very different and that makes it difficult, if possible, to make firm conclusions on the issues such as optimal training volumes and types of MIT intervention.

## Conclusion

In general, the current review shows that MIT has better estimated effects on enhancing muscle strength compared to no exercise, but inferior to PT. The intervention of the combination of MIT and PT is equivalent to PT alone in enhancing MVC force. The subgroup group analyses further suggest that older adults and small finger muscles may be beneficial more from MIT than young adults and larger muscles. In summary, the current review suggests that MIT is an effective substitute or addition to PT in muscle strength training. Therefore, MIT should be considered and applied in rehabilitation settings when physical training is too demanding for patients with motor disabilities (e.g., poststroke rehabilitation) or high-intensity resistance training is impractical or venerable to injuries (e.g., very old adults).

## Data availability statement

The original contributions presented in this study are included in the article/supplementary material, further inquiries can be directed to the corresponding authors.

## Author contributions

XL, GY, and WY: conception, design of study, and acquisition of data. XL, GY, WY, SG, AC, and ZY: analysis and/or interpretation of data and drafting the manuscript. All authors contributed to the article and approved the submitted version.
